# Creep of Concrete in Shell Structures: Nonlinear Theory

**DOI:** 10.3390/ma16165587

**Published:** 2023-08-11

**Authors:** Turlybek Turkpenovich Mussabayev, Zhmagul Smagulovich Nuguzhinov, Darya Nemova, Tabyldy Kayupov, Temirkhan Anapiyaevich Tolkynbaev, Assel Zhanalykovna Akmakanova, Gulzhan Sailaubekovna Khafizova

**Affiliations:** 1 Construction Department, L.N. Gumilyov Eurasian National University, Kazhymukan 13, Astana 010000, Kazakhstan; kayupov_t@enu.kz (T.K.); tolkynbayev_ta@enu.kz (T.A.T.); akmakanova_azh_2@enu.kz (A.Z.A.); khafizova_gs_1@enu.kz (G.S.K.); 2 KazMIRD, Karaganda State Technical University, Nursultan Nazarbayev Ave. 56/6, Karaganda 100027, Kazakhstan; kazmirr@mail.ru; 3 Laboratory of Protected and Modular Structures, Peter the Great St. Petersburg Polytechnic University, 195251 Saint Petersburg, Russia

**Keywords:** theory of cracks, concrete creep, nonlinear theory of calculation of structures, building codes

## Abstract

The creep of concrete is one of the main problems threatening concrete structural development and the stability and safety of structures. However, the nonlinear theory is the key to solving the problem of taking into account the physical and mechanical properties of concrete creep in shell structures. To create such a theory, the original shell is replaced by a continuous equivalent elastic shell. To determine the stress–strain state of the structure, the equations of nonlinear creep and crack growth are derived, and a deformation model of the section is created. The behavior of the structure at all stages of the life cycle is investigated by solving the solving systems of differential equations of equilibrium, motion, and perturbation of the equivalent shell. The values of the ratios of dependence of long-term and short-term critical loads on deformations, forces, cracks, etc., are given. The accuracy of the solution of the developed nonlinear theory is compared with the linear theory of concrete creep as well as experimental data. The results show that, according to the linear theory, for the values for the short term and long term, up to 56% and up to 39% of critical loads are overestimated, respectively. The creep process in practical engineering can be effectively controlled by the results of the proposed theory.

## 1. Introduction

The phenomenon of metal creep at high temperatures is one of the reasons for the destruction of the Twin Towers in New York (USA) [1]. Due to the creep of materials, the structures of Basmanny Market in Moscow (Russia) and a bridge in Genoa (Italy) collapsed [2].

The development of creep increases the stress loss in the concrete structure and redistributes the internal force of statically indeterminate structures. In turn, excessive deformation of the structure significantly reduces the overall strength and even causes loss of bearing capacity. With the modern, wide range, high-quality, and common use of concrete materials, concrete creep has become one of the main problems threatening structural composition, the quality characteristics of concrete, as well as the safety and long-term stability of concrete structures [3,4,5].

The creep of concrete elements can weaken the greatest tensile stress, which reduces the risk of the early cracking of concrete [6,7,8,9,10,11,12]. Evaluation of the crack resistance of concrete should take into account the creep of concrete [13].

Taking into account the creep of concrete under axial compression in calculations within the framework of the classical theoretical approach was first proposed by A.R. Rzhanitsyn and subsequently developed by Y.N. Rabanov, S.A. Shestikov, L.B. Bunyatyan, V.B. Kolmanovsky, I.E. Prokopovich, E.A. Yatsenko, and I.I. Ulitsky for linear creep problems of concrete [14,15,16,17,18,19,20,21,22]. Distefano I.N., Prokopovich I.E., and Linnik V.S. applied this method using the nonlinear creep equation of Harutyunyan N.H. and two significantly simplified assumptions: an idealized section in the form of two thin strips; in the function of the nonlinear creep of concrete, the stress was considered to be the same for both bands (when bending the section) [20,23,24]. Such a simplified model in a narrow range allows us to obtain in the final form qualitative nonlinear estimates of the limit of long-term stability.

Creep deformation has a linear dependence on the applied stress in the case of a low stress level (compressive strength less than 30%). However, creep is an unstable phenomenon, since time and stress increase in the case of a high stress level (from 30% to 80% compressive strength); that is, it is a nonlinear dependence that is found in specific creep theories and experimental research data [25,26,27,28,29].

A review of the literature shows that the development of effective unified computational algorithms for solving physically nonlinear problems of the dynamics of short-term and long-term deformation of shells and plates made of composite materials is an urgent problem today [30,31].

The value of long-term resistance $R_{i}$ is established on the basis of local experiments and depends on the choice of empirical correction coefficients:

(1) $$R_{l}=\frac{\pi^{2}\cdot E\cdot I}{L^{2}\left(1+\varphi_{\infty }\right)},$$

where $R_{l}$ is long-term resistance (by Rzhanitsin) [14], E is a modulus of the deformation of construction materials, I is the moment of inertia of the section of the structure, *L* is the length of the column for the core elements, and $\varphi_{\infty }$ is the standard normalised dimensionless creep characteristic of concrete.

The value of long-term resistance contains deviations from the results. With the prolonged action of the load, the empirical method dangerously overestimates the conditional critical force by several times concerning the value of the limiting elasticity of concrete. Formula (2) shows the modulus of deformation of concrete and the numerical values of the ultimate creep characteristic $\varphi_{b,cr}$:

(2) $$E_{b,\tau }=\frac{E_{b}}{1+\varphi_{b,cr}},$$

where $\varphi_{b,cr}$ is the concrete creep coefficient.

**Figure 1:**
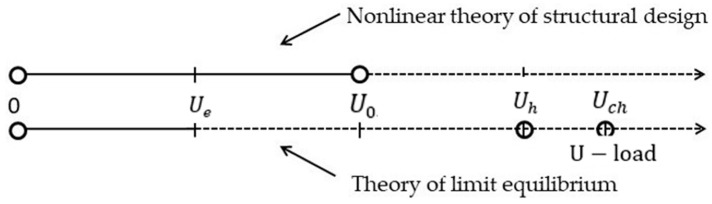
Theories for calculating the limit state of the bearing capacity of structures: $U_{e\;}\;$ is the elastic behavior of the structure, $U_{0}\;$ is the limit state of the structure, $U_{h}\;$ is a plastic hinge in the design, and $U_{ch}\;$ is the corrected plastic hinge.

The principles of the general nonlinear theory of the calculation of structures are the basis of European standards [29,32]. Figure 1 shows that according to this concept, on the top line of the diagram, the design is considered to be fully compliant with safety requirements until the load E, increasing continuously from a point 0, will not reach a certain level $E_{0}$, beyond which the design does not meet the safety requirements.

The theory of limit equilibrium excludes the indicator $U_{0}$ from the design scheme of the limit state of a compressed reinforced concrete structure [33]. This concept is contrary to the principles of the nonlinear theory of the calculation of structures and is shown on the bottom line of the diagram. The limiting state of the structure is assigned by a volitional method at a point $U_{h}>U_{0}$ and corresponds to the formation of a plastic hinge ${(U=U}_{h})$. The value $U_{h}$ may be several times greater than the value ${\;U}_{0}$. The process of continuous static loading of the structure is carried out only on the interval (0, $U_{e}$) of the elastic operation of the structure. At the point $U_{e}$, either the first crack in the concrete is formed or plastic deformation begins. From point $U_{e}$ in norms, there is an instantaneous jump to the point ${\;U}_{h}$, bypassing the point ${\;U}_{0}$, or to the point $U_{ch}$, corresponding to the plastic hinge.

Contradictions between the limit state of the structure, established by the nonlinear theory of the calculation of structures, and the limit state are presented in the building codes:

1. Point $U_{0}$—there is a real point completing the process of static loading of a compressed reinforced concrete structure; point $U_{h}$—there is an unreal point, but it is used in the building codes and has nothing to do with the process of static loading of any compressed reinforced concrete structure;
2. The nonlinear theory of the calculation of structures recognizes the unsatisfactory state of the structure, corresponding to the site ($U_{0},U_{h})$; building codes recognize the same deformation site $\left(U_{0},U_{h}\right)$ satisfactory condition for construction;
3. The nonlinear theory of the calculation of structures establishes the rules for calculating the value${\;U}_{0}$ and characterizes the limiting state of the structure;
4. The nonlinear theory of calculating structures naturally rejects the existence of a deflection at the column, with it having no length; the building codes give deflection to the column, with it having no length. This deflection reaches infinite values in absolute magnitude;
5. Numerical values $U_{0}$ and $U_{h}$ differ from each other up to 100%.

The review shows that the existing theories of the reinforced concrete shell have significant shortcomings.

The nonlinear theory of concrete creep within the framework of instantly elastic models has been developed in scientific works [34,35,36].

The nonlinear creep equation for concrete, which describes the biaxial stress state, has the form [34]:

(3) $$\begin{array}{l} \varepsilon_{11}\left(t\right)=\frac{\sigma_{11}\left(0\right)-\nu \cdot \sigma_{22}\left(0\right)}{E\left(0\right)}\cdot \left[1+f\left(\sigma_{i}\right)\cdot \varphi \left(t\right)\right]\\ \;+\int_{0}^{t}\left\{\begin{array}{c} \frac{d}{d_{\tau }}\left[\sigma_{11}\left(\tau \right)-\nu \cdot \sigma_{22}\left(\tau \right)\right]\cdot \\ \cdot \left(\frac{1}{E\left(\tau \right)}+\frac{\varphi \left(t\right)-\varphi \left(\tau \right)}{E\left(0\right)}\cdot f\left[\sigma_{i}\left(\tau \right)\right]\right) \end{array}\right\}\cdot d\tau ; \end{array}\;\begin{array}{l} \varepsilon_{22}\left(t\right)=\frac{\sigma_{22}\left(0\right)-\nu \cdot \sigma_{11}\left(0\right)}{E\left(0\right)}\cdot \left[1+f\left(\sigma_{i}\right)\cdot \varphi \left(t\right)\right]\\ +\int_{0}^{t}\left\{\frac{d}{d_{\tau }}\left[\sigma_{22}\left(\tau \right)-\nu \cdot \sigma_{11}\left(\tau \right)\right]\cdot \left(\frac{1}{E\left(\tau \right)}+\frac{\varphi \left(t\right)-\varphi \left(\tau \right)}{E\left(0\right)}\cdot f\left[\sigma_{i}\left(\tau \right)\right]\right)\right\}\cdot d\tau ; \end{array}\;\begin{array}{c} \gamma_{12}\left(t\right)=\frac{2\cdot \left(1+\nu \right)}{E\left(0\right)}\cdot \tau_{12}\left(0\right)\cdot \left[1+f\left(\sigma_{i}\right)\cdot \varphi \left(t\right)\right]\\ \;+\int_{\tau_{1}}^{t}2\left(1+\nu \right)\cdot \left\{\frac{d\tau_{12}\left(\tau \right)}{d_{\tau }}\cdot \left(\frac{1}{E\left(\tau \right)}+\frac{\varphi \left(t\right)-\varphi \left(\tau \right)}{E\left(0\right)}\cdot f\left[\sigma_{i}\left(\tau \right)\right]\right)\right\}\cdot d\tau . \end{array}$$

where φ(t) is the current value of the creep characteristic, *E*(0) *is* the modulus of deformations at the initial moment of time, and $f(\sigma_{i})$ is the nonlinearity function, which is selected based on the experimental data depending on the grade of the concrete and other factors.

The equation of the nonlinear creep of concrete has the form [35]:

(4) $$\varepsilon \left(t,t_{0}\right)=S_{0}\left[\sigma \left(t\right)\right]\cdot \left\{\sigma \left(t\right)\left[\frac{1}{E^{M}\left(t\right)}+C_{0}^{*}\left(t,t_{0}\right)-\int_{t_{0}}^{t}\sigma \left(\tau \right)\cdot \frac{𝜕}{𝜕\tau }C_{0}^{*}\left(t,\tau \right)\cdot d\tau \right]\right\}$$

where $\varepsilon \left(t,\;t_{0}\right)\;$ is the total deformation of concrete under the action of constant stress applied at time $t_{0}$, $S_{0}[d\sigma^{\prime }(t)]$ is the nonlinearity function of instantaneous deformation, σ(t) is alternating stress, and $E^{M}(t)$ is the modulus of instantaneous deformation. Deformations at time t, $C_{0}^{*}\left(t,\;t_{0}\right)\;и\;C_{0}^{*}\left(t,\;t\right)$ (Equation (6)) are creep measures at the time of loading and observation in the form of N. Harutyunyan’s record [37]. $C_{0}^{*}\left(t,\;\tau \right)\;$ is a creep measure in the form proposed by S. Alexandrovsky [38].

The equation of nonlinear creep of concrete [36] for the first time takes into account the nonlinearity of the instantaneous deformation of concrete in the framework of heredity hypotheses:

(5) $$\varepsilon \left(t\right)=\frac{\sigma \left(t\right)}{E\left(t\right)}\cdot F_{M}\left[\sigma \left(\tau \right)\right]-\int_{t_{0}}^{t}\sigma \left(\tau \right)\cdot F_{n}\left[\sigma \left(\tau \right)\right]\cdot \frac{𝜕}{𝜕\tau }C_{0}^{*}\left(t,\tau \right)\cdot d\tau .$$

where $\varepsilon \left(t\right)$ is the relative creep deformation of the material at time t, *E*(*t*) is the modulus of elastic-instantaneous deformations, $F_{M}$ is a nonlinear stress function for elastic-instantaneous deformations, $\sigma \left(\tau \right)\;$ is an alternating stress, and $F_{n}\;$ is a nonlinear stress function for creep deformations.

The nonlinear creep of concrete [36] is written as follows:

(6) $$\varepsilon \left(t,t_{0}\right)=S_{0}\left[\sigma \left(t\right)\right]\cdot \left\{\sigma \left(t\right)\cdot \left[\sigma \left(t\right)\cdot \frac{1}{E^{M}\left(t\right)}+C_{0}^{*}\left(t,t\right)-\int_{t_{0}}^{t}\sigma \left(\tau \right)\cdot \frac{𝜕}{𝜕\tau }C_{0}^{*}\left(t,\tau \right)\cdot d\tau \right]\right\}.$$

The equation of the nonlinear creep of concrete takes into account the nonlinearity of the instantaneous deformation of concrete and has the form [39]:

(7) $$\begin{array}{l} {\dot{\varepsilon }}_{b}\left(t\right)=\frac{1}{E_{b}\left(t\right)}\left[\frac{𝜕\Phi \left[\sigma_{b},t\right]}{𝜕\sigma_{b}\left(t\right)}{\dot{\sigma }}_{b}\left(t\right)+\frac{𝜕\Phi \left[\sigma_{b},t\right]}{𝜕t}\right]+\frac{𝜕f\left[\Phi \left(\sigma_{b},t\right)\right]}{𝜕\Phi }\varphi_{t}^{\Phi }\alpha_{\Phi }\cdot \\ \left[\frac{𝜕\Phi \left[\sigma_{b},t\right]}{𝜕\sigma_{b}\left(t\right)}{\dot{\sigma }}_{b}\left(t\right)+\frac{𝜕\Phi \left[\sigma_{b},t\right]}{𝜕t}\right]+\frac{1}{E_{b0}}\cdot {\dot{\varphi }}_{t}^{\Phi }f\left[\Phi \left(\sigma_{b},t\right)\right]. \end{array}$$

where ${\dot{\varepsilon }}_{b}(t)$ is the creep deformation rate of concrete at time *t*, $E_{b}(t)$ is the concrete deformation modulus for the current time, ${\dot{\sigma }}_{b}(t)$ is the rate of stress change in concrete at time *t*, $\varphi_{t}^{\Phi }\;$is the creep characteristic of concrete in the form of a Sanzharovsky [34] notation, $\alpha_{\Phi }$ is a constant in the form of a Sanzharovsky notation, and $E_{b0}\;$is the modulus of elasticity.

A general theory of calculation of composite physically and geometrically nonlinear thin-walled systems is proposed, extending to a fairly wide class of smooth and reinforced shell and plate structures, including during their reconstruction, operating in a complex stress state at real loading levels, as well as under conditions of nonlinear creep of the material and the presence of cracks.

A mathematical model of the behavior of the mentioned structures at all stages of loading is constructed within the framework of unified systems of differential equations and an algorithm for their solution at different points in time.

A new method for calculating inelastic shell structures with cracks is proposed, and relations for equivalent elasticity parameters are given, which implement quite strictly the problem of taking into account plasticity, half-strength, and cracks in stretched and compressed cross-section zones. The main idea is to replace an inelastic shell with cracks—a solid equivalent elastic shell structure with equivalent elasticity parameters determined by comparing its deformations with similar deformations of a nonlinear model.

## 2. Methods

Building codes ensure that the general criteria and design methods comply with the requirements of the nonlinear theory of structural analysis.

Practical adaptation requires overcoming existing inconsistencies in the building codes, the calculated position of which is based on an erroneous model of a plastic hinge [40,41,42]. In the nonlinear theory of structures, the calculation of structures is presented in a simple and convenient form; it corresponds to the deformation model of the section. This measure will allow finding alternative solutions in the design of various structures.

The construction of a nonlinear theory for the calculation of reinforced concrete shells and plates based on generally accepted assumptions is shown. The well-known models of concrete deformation under short-term and long-term loading are considered, disobedient to Hooke’s law σ=E·ε.

The nonlinear relationship between stresses and deformations was first considered by G. Bulfinger and F. Gerstner. The power law at k≠1 is a nonlinear dependence, which is written in the following form (8):

(8) $$\sigma =A\cdot \varepsilon^{k},$$

where A is a constant with the dimension of the stresses and k is an exponent (dimensionless quantity).

The following equations are also used to describe the nonlinear relationship between stresses and strains.

F. Gerstner describes the nonlinearity of materials by parabolic dependence:

(9) $$\sigma_{b}=A_{1}\cdot \varepsilon -A_{2}\cdot \varepsilon^{2}.$$

The functional relationship between stress intensity and strain intensity in the material deformation diagram $\sigma_{\varepsilon }$ is approximated by a cubic dependence [43] in the form:

(10) $$\sigma_{i}=E_{0}\cdot \varepsilon_{i}-A_{3}\cdot \varepsilon_{i}^{3}.$$

The polynomial function is taken as:

(11) $$\sigma_{b}=A_{1}\cdot \varepsilon_{b}+A_{2}\cdot \varepsilon_{b}^{2}+A_{3}\cdot \varepsilon_{b}^{3}+A_{4}\cdot \varepsilon_{b}^{4}+A_{5}\cdot \varepsilon_{b}^{5}.$$

Within the framework of the hypothesis of the linear creep of concrete, the creep of particular importance is in the form of:

(12) $$\frac{\sigma \left(t\right)}{E\left(t\right)}=\varepsilon \left(t\right)+\int_{t_{0}}^{t}\varepsilon \left(\tau \right)\cdot R\left(t,\tau \right)\cdot d\tau .$$

The simplified Maxwell–Kachanov concrete creep formula is taken in differential form:

(13) $$\dot{\varepsilon }=B_{1}\left(t\right)\cdot \sigma^{m}+\frac{1}{E}\cdot \dot{\sigma }.$$

Next, the problems of constructing a nonlinear theory for the calculation of reinforced concrete shells and plates are considered.

## 3. Results and Discussion

### 3.1. Establishing a Functional Relationship between Stresses and Strains

In the first stage, a deformed thin-walled element and its cross-section of unit length are considered, receiving displacements with these U,V,W rotation angles, this $\omega_{1},\omega_{2},\omega_{3},$ elongation strain, and these $\varepsilon_{1},\varepsilon_{2},$ longitudinal γ and transverse $\gamma_{1},\gamma_{2}$ shifts [44].

The hypotheses of the theory of plasticity are accepted, and specific equations of fiber deformation in the differential form are obtained with increasing load for the case of short-term loading in the form:

(14) $${\dot{\sigma }}_{11}\left(t\right)=E_{1}^{*}\cdot {\dot{\varepsilon }}_{11}\left(t\right)+E_{2}^{*}\cdot {\dot{\varepsilon }}_{22}\left(t\right)+E_{3}^{*}\cdot {\dot{\gamma }}_{12}\left(t\right)+E_{4}^{*}\cdot {\dot{\gamma }}_{13}\left(t\right)+E_{5}^{*}\cdot {\dot{\gamma }}_{23}\left(t\right);\ldots ;\;{\dot{\tau }}_{12}\left(t\right)=E_{11}^{*}\cdot {\dot{\varepsilon }}_{11}\left(t\right)+E_{12}^{*}\cdot {\dot{\varepsilon }}_{22}\left(t\right)+E_{13}^{*}\cdot {\dot{\gamma }}_{12}\left(t\right)+E_{14}^{*}\cdot {\dot{\gamma }}_{13}\left(t\right)+E_{15}^{*}\cdot {\dot{\gamma }}_{23}\left(t\right);\ldots ;$$

where $E_{j}^{*}\left[\sigma_{ij}\left(t\right)\right]$ are variable modules of deformations, $E_{c}\left[\sigma_{ij}\left(t\right)\right]$ are secant modules, and $E_{k}\left[\sigma_{ij}\left(t\right)\right]$ are tangent modules of deformations from the concrete deformation diagram $\sigma_{ij}∼\varepsilon_{ij}$.

For research on the processes of the creep of materials in structures, any of the equations of creep theories are used; exactly, equations of the theory of ageing in differential form, which have the form:

(15) $${\dot{\varepsilon }}_{jj}\left(t\right)=\frac{1}{E\left(t\right)}\cdot \left[{\dot{\sigma }}_{jj}\left(t\right)+\vartheta {\overset{˙}{\cdot \sigma }}_{\left(3-j\right)\left(3-j\right)}\left(t\right)\right]+\frac{1}{E\left(0\right)}\cdot \left[\sigma_{jj}\left(t\right)+\vartheta \cdot \sigma_{\left(3-j\right)\left(3-J\right)}\left(t\right)\right]\cdot f\left[\sigma_{i}\left(t\right)\right]\;\cdot \dot{\varphi }\left(t\right);\;{\dot{\gamma }}_{12}\left(t\right)=\frac{2\cdot \left(1+\vartheta \right)}{E\left(t\right)}\cdot {\dot{\tau }}_{12}\left(t\right)+\frac{1}{E\left(0\right)}\cdot \tau_{12}\left(t\right)\cdot f\left[\sigma_{i}\left(t\right)\right]\cdot \dot{\varphi }\left(t\right);\;{\dot{\gamma }}_{j3}\left(t\right)=\frac{2\cdot \left(1+\vartheta \right)}{E\left(t\right)}\cdot {\dot{\tau }}_{j3}\left(t\right)+\frac{1}{E\left(0\right)}\cdot \tau_{j3}\left(t\right)\cdot f\left[\sigma_{i}\left(t\right)\right]\cdot \dot{\varphi }\left(t\right),$$

where $E\left(\tau \right)=\frac{E_{c}\left(\tau \right)}{\left[1+g\left(\tau \right)\right]};\;g\left(\tau \right)=\frac{E_{c}\left(\tau \right)\cdot \left(0.5-\vartheta_{0}\right)}{1.5E_{0}};\;f\left[\sigma_{i}\left(\tau \right)\right]$ is the nonlinearity function; and $\varphi \left(t\right)$ is a creep characteristic.

The combination of these two laws of short-term and long-term deformation is applicable at all stages of the structure’s existence.

Geometric relations are obtained based on a Timoshenko-type shift model:

(16) $$\varepsilon_{jj}=\varepsilon_{j}+z\cdot \frac{𝜕\psi_{j}}{𝜕j};\;\gamma_{12}=\gamma +2z\cdot \left(\frac{𝜕\psi_{1}}{𝜕y}+\frac{𝜕\psi_{2}}{𝜕x}\right);\;\gamma_{j3}=\gamma_{j}\cdot f\left(z\right);\;\gamma_{j}=\psi_{j}+\frac{𝜕W}{𝜕j}-\frac{U}{R_{j}};\;j=x,y;\;U↔V.$$

Transverse shear stresses are distributed according to the parabola law in the following form:

(17) $$\tau_{j3}=-\frac{Q_{j}^{BH}}{z_{mj}\left(t\right)+z_{oj}\left(t\right)}\cdot f\left(z\right);\;f\left(z\right)=-\frac{6}{{\left[z_{mj}\left(t\right)+z_{oj}\left(t\right)\right]}^{2}}\cdot \left[z+z_{oj}\left(t\right)\right]\left[z-z_{mj}\left(t\right)\right].$$

In this case, the following conditions are met:

(18) $$\int_{-z_{oj}\left(t\right)}^{z_{mj}\left(t\right)}f\left(z\right)\cdot dz=\left[z_{mj}\left(t\right)+z_{oj}\left(t\right)\right];\;\frac{1}{z_{mj}\left(t\right)+z_{oj}\left(t\right)}\int_{-z_{oj}\left(t\right)}^{z_{mj\left(t\right)}}f^{2}\left(z\right)\cdot dz=\frac{1}{k_{sh}},$$

where $k_{sh}$ is the shape coefficient at shift and $z_{oj}\left(t\right),z_{mj}\left(t\right)$ are the upper and lower limits of integration within the solid part of the section, depending on the presence or absence of cracks.

The shape coefficient at shift is derived to obtain shear stresses at the center of gravity of the shell cross-section with cracks, depending on the shape of the section.

### 3.2. Creation of a Deformation Model of a Section with Cracks and the Inelastic Properties of Materials

**Figure 2:**
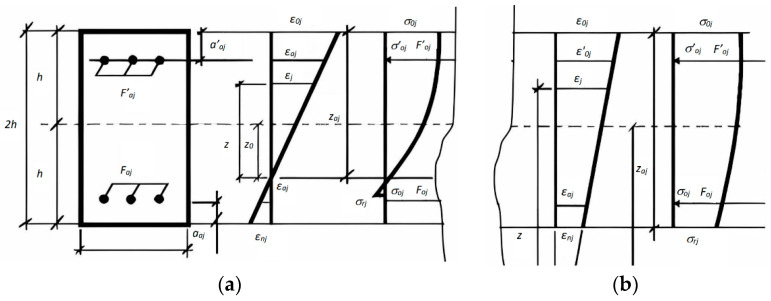
Epure of the distribution of stresses and strains in the section of the element. (**a**) There are cracks in the section. (**b**) There is no a crack in the section.

The main vector and the main moment of the stress diagram in a linear section are compiled in the form:

(19) $$N_{j}^{BH}\left(t\right)=\int_{-z_{oj}\left(t\right)}^{z_{mj}\left(t\right)}\sigma_{jj}\left(t\right)\cdot dz-\sum_{k=1}^{n^{\prime }}\sigma_{akj}^{\prime }\left(t\right)\cdot F_{akj}^{\prime }+\sum_{k=1}^{n}\sigma_{akj}\left(t\right)\cdot F_{akj}\ldots ;\;{\;Q}_{j}^{BH}\left(t\right)=\int_{-z_{oj}\left(t\right)}^{z_{mj}\left(t\right)}\tau_{j3}\left(t\right)\cdot dz+\sum_{k=1}^{n^{\prime }}\tau_{akj3}^{\prime }\left(t\right)\cdot F_{akj}^{\prime }+\sum_{k=1}^{n}\sigma_{akj3}\left(t\right)\cdot F_{akj};\;{\;M}_{j}^{BH}\left(t\right)=\int_{-z_{oj}\left(t\right)}^{z_{mj}\left(t\right)}\sigma_{jj}\left(t\right)\cdot z\cdot dz+\sum_{k=1}^{n^{\prime }}\sigma_{akj}^{\prime }\left(t\right)\cdot F_{akj}^{\prime }\cdot h\prime_{akj}\;+\sum_{k=1}^{n}\sigma_{akj}\left(t\right)\cdot F_{akj}\cdot h_{akj};{\;z}_{mj}\left(t\right)=\frac{h_{mj}\cdot \left(\varepsilon_{pj}-\varepsilon_{j}\right)}{\varepsilon_{mj}-\varepsilon_{j}};\;z_{oj}\cdot \left(t\right)=\frac{h_{oj}\cdot \left(\varepsilon_{rj}-\varepsilon_{j}\right)}{\varepsilon_{oj}-\varepsilon_{j}};\ldots ;\;z_{m}\left(t\right)=\frac{h_{m}\cdot \left(\varepsilon_{rj}-\gamma \right)}{\gamma_{0}-\gamma },$$

where $\varepsilon_{oj}$, $\varepsilon_{mj}\left(j=1,2\right)$, $\gamma_{0}$, $\gamma_{m}$ are fiber deformations of the cross-section; $\sigma_{jj},$ $\tau_{12},$ $\tau_{j3},$ are the linear stresses in the concrete matrix; $\sigma \prime_{aj},\ldots ,\sigma_{aj},\ldots uF_{aj}^{\prime },F_{aj}$ are, stresses and total areas j upper and lower reinforcing bars, respectively; $h\prime_{aj},h_{aj}$ are coordinates of the reinforcing bars of the location relative to the center of reduction of the internal forces of the section’ and $a\prime_{aj},a_{aj}$ is the thickness of the upper and lower protective layers of concrete. To simplify the epure of the distribution of stresses and strains k, there is an amount of upper and lower layers of reinforcement. To represent the distribution of stresses and strains, Figure 2 shows the location and number of top and bottom reinforcing bars in the structure section.

Figure 2 shows the diagrams of the distribution of deformation and stress in the section of the element: (a) if there are cracks in the section and (b) if there are no cracks in the section.

The fixed ultimate deformations in the section are taken subject to the strength condition in the form:

(20) $$\varepsilon_{lj}=\left\{\begin{array}{l} \varepsilon_{kj}providedn_{cond}\neq 0orn_{cond}=0;\\ \varepsilon_{kj}^{*},\;\varepsilon_{jj}^{*}\leq \varepsilon_{j\left(3-j\right)}^{*}\leq \varepsilon_{\left(3-j\right)\left(3-j\right)}^{*}<0;\\ \varepsilon_{jj}^{*},\;\varepsilon_{jj}^{*}\leq \varepsilon_{j\left(3-j\right)}^{*}\leq \varepsilon_{\left(3-j\right)\left(3-j\right)}^{*}>0; \end{array}\right.\;\left[\left(k\right),l=r,p,\left(o,m\right);\;j=1,2,\;1↔2\right],\;\varepsilon_{\left(3-j\right)\left(3-j\right)}^{*}=\varepsilon_{\left(3-j\right)j}^{*}\left(j=1↔2\right),$$

where $\varepsilon_{jj}^{*}\left(j=1,2\;1↔2\right)$ are fixed ultimate deformations of the composite matrix in tension and compression and $n_{cond}$ are conditions for the strength of the Equation (21).

With numerical integration, the boundary of the occurrence of plastic deformations in the reinforcement corresponds to the achievement of yield strains $\varepsilon_{m}$ on the reinforcement deformation diagram $\sigma_{a}\sim \varepsilon_{a}$.

When creating a deformation model of the cross-section, the condition for the appearance of a crack during compression and tension was adopted in the form of a condition (criterion) of strength by G. Geniev (21) [45]:

(21) $$\sigma_{r1}^{2}+\sigma_{r2}^{2}+\sigma_{r3}^{3}-\left(\sigma_{r1}\cdot \sigma_{r2}+\sigma_{r2}\cdot \sigma_{r3}+\sigma_{r1}\cdot \sigma_{r3}\right)-\left(R_{cs}-R_{ts}\right)\cdot \left(\sigma_{r1}+\sigma_{r2}+\sigma_{r3}\right)\;-R_{cs}\cdot R_{ts}=0,$$

where $R_{cs}\;and\;R_{ts}$ are compressive and tensile strength, respectively.

To relate the stresses and strains in the fibers, there are equations of the forms (14) or (15). The specific equilibrium equations in the cross-section with cracks are compiled in differential form. A linear system of differential equations with time-varying coefficients is obtained in the form:

(22) $$\left[A\left(x,t\right)\right]\times \left\{\dot{x}\right\}=\left\{A_{p}\left(t,x\right)\right\},$$

where $\left[A\left(x,t\right)\right]$ is a square matrix of coefficients with an unknown system, the order of which depends on the accepted model, and $\left\{\dot{x}\right\}$ is a vector column of unknown velocities; $\left\{A_{p}\left(t,x\right)\right\}$; there is a matrix column of free terms.

The solution of Equation (22) makes it possible to find edge deformations with the help of which all parameters of the stress–strain state of the section are determined.

### 3.3. Derivation of the Integral Relationship between Deformations and Stresses, and Finding Equivalent Elasticity Parameters

Equivalent elastic parameters are obtained based on te calculated edge deformations of the section.

To achieve this, m—the moment relationship between the total deformation of an arbitrary layer—is expressed with a coordinate $z+z_{0}$. This is how the equivalent elasticity parameters are found for different models of a section with cracks and inelastic properties of materials:

(23) $$\begin{array}{c} \;\frac{1}{2}\int_{-h}^{h}\varepsilon_{mr}\left(1+\frac{1}{h}\right)+\varepsilon_{or}\left(1-\frac{1}{h}\right){\left(z+z_{0}\right)}^{m}dz=\\ =\int_{-h}^{h}\left[\varepsilon_{1}\cdot L^{2}+\varepsilon_{2}\cdot n^{2}+\gamma \cdot L_{n}+z\cdot \left(\frac{𝜕\psi_{1}}{𝜕x}\cdot L_{k}^{2}+\frac{𝜕\psi_{2}}{𝜕y}\cdot n_{k}^{2}+\left(\frac{𝜕\psi_{1}}{𝜕y}+\frac{𝜕\psi_{1}}{𝜕x}\right)L_{k}n_{k}\right)\right]{\left(z+z_{0}\right)}^{m}dz, \end{array}$$

where $L=\sin\alpha ;\;n=\cos\alpha ;\;\alpha =tg^{-1}\left[\frac{\gamma }{\left(\varepsilon_{2}-\varepsilon_{1}\right)}\right];\;L_{k}=\sin\beta ;\;n_{k}=\cos\beta ;\;\beta =tg^{-1}\left[2\chi /(\chi_{2}-\chi_{1}\right]$. Expressions for elongation, shift, and curvature from the elastic calculation are substituted in the right part of Formula (23), and integration is performed. As a result, equivalent elasticity parameters are found for different models of a section with cracks and the inelastic properties of materials, in particular, for a model of the Timoshenko type:

(24) $$E_{eq}=\frac{N_{r1}+M_{r1}-0.5\cdot \left(N_{r2}+M_{r2}-6k_{sh}\cdot Q_{r,r}\right)}{\frac{\varepsilon_{mr}\left(h+V_{r}\right)-\varepsilon_{or}\left(-h+v_{r}\right)}{2h}\cdot F\cdot S_{r}-\frac{1-2v_{0}}{2E_{0}}\cdot \left(N_{r2}+M_{r2}-2k_{sh}\cdot Q_{r,r}\right)};\;v_{eq}=\frac{1}{2}-\left(\frac{1}{2}-v_{0}\right)\cdot \frac{E_{eq}}{E_{0}}.\;FS_{r}=\frac{\left[{\left(h+z_{0}\right)}^{m+1}-(-h+z_{0})\right]}{m+1};\;v_{r}=\frac{S\cdot I_{r}}{F\cdot S_{r}};\;m=\frac{1-\frac{\varepsilon_{m1}}{\varepsilon_{01}}}{2};\;SI_{r}=\left[{\left(h+z_{0}\right)}^{m+1}+{\left(-h+z_{0}\right)}^{m+1}\right]\cdot \frac{h}{m+1}-\frac{\left[{\left(h+z_{0}\right)}^{m+2}-{\left(-h+z_{0}\right)}^{m+2}\right]}{\left(m+1\right)\cdot \left(m+2\right)},$$

where $N_{rj,}M_{rj,}Q_{r,r}$ are the internal forces in the principal axes; $F_{nj},I_{nj}$ are the reduced characteristics of the reinforced concrete section; $F\cdot S_{r},S\cdot I_{r}$ are the integral geometric characteristics of the section; and m is an indicator of the weight of the influence of the extreme fibers of the section.

As a result of the integral representation of material properties (24), physical equations of a nonlinear model with load-dependent parameters E,v can be replaced by physical relations with equivalent elasticity parameters.

### 3.4. Description of the Shape of the Deformed Scheme and Nonlinear Analysis of Structures

The shell calculation model is constructed according to a deformed scheme based on differential equilibrium equations.

The features of deformation and the existence of the shell are accounted for. Functional dependencies of changes in external influences and/or the rheological properties of materials corresponding to the real process of loading and deformation are set:

(25) $$\frac{𝜕N_{1}}{𝜕x}+\frac{𝜕N_{12}}{𝜕y}+N_{2}\left(\frac{𝜕\omega_{m}}{𝜕y}\right)+N_{12}\left(\frac{𝜕\omega_{m}}{𝜕x}\right)-q_{1}\left(1+\varepsilon_{1}+\varepsilon_{2}\right)-q_{2}\left(\omega_{p}+\omega_{m}\right)=0;\;\left(1↔2\right),$$

where $\frac{𝜕M_{1}}{𝜕x}+\frac{𝜕M_{12}}{𝜕y}-Q_{1}=0;\;\frac{𝜕M_{12}}{𝜕x}+\frac{𝜕M_{2}}{𝜕y}-Q_{2}=0.$ To trace the entire loading process, the law of increasing load is presented in the form:

(26) $$q\left(t,x,y\right)=q_{0}\left(x,y\right)+\Delta q\left(x,y\right)\cdot t.$$

A resolving system of quasi-static equations of shell motion due to changes in external load or geological processes is constructed. All relations used are differentiated once in time, including physical relationships with equivalent elasticity parameters, taking into account variability. As a result, a linear system of differential equations is obtained concerning the generalized velocities of the deformation parameters:

(27) $$\left\{{\dot{y}}_{J}\left(t\right)\right\}\cdot \left[a_{jj}\left(t\right)\right]=a_{JP}\left(t\right),$$

where$\;{\dot{y}}_{J}\left(t\right)$ is the generalized velocities of the deformation components; ${\;a}_{JJ}\left(t\right)$ is the variable coefficients, depending on the condition and age of the structure; and $a_{JP}\left(t\right)$ is variable functions of changing the external load and/or creep parameters of materials.

### 3.5. Evaluation of the Stability of the Equilibrium under Study and the Establishment of the Criterion for the Loss of Bearing Capacity

To check the stability of the equilibrium of the structure, its varied state is considered. A system of linear equations in variations of the desired quantities is derived. As a result, a homogeneous system of linear equations in variations is obtained with coefficients recalculated at each calculation step:

(28) $$\left\{\delta y_{J}\left(t\right)\right\}\cdot \left[a_{JJ}\left(t\right)\right]=0.$$

The equality of the determinant of this system to zero determines the condition for the loss of stability of the equilibrium of the structure.

(29) $$Det\left[a_{JJ}\left(t\right)\right]=0,$$

where $\delta y_{J}\left(t\right)is$ the perturbation components and $a_{JJ}\left(t\right)is$ the virtual equivalent stiffnesses when the structure is perturbed.

The proposed mathematical model and algorithm for solving nonlinear problems are implemented in the form of a compiled set of computer programs. The developed software systems have been tested on solving test problems, with satisfactory comparison of shell calculation results with known solutions [46,47] and experimental data [48,49]. They passed the state registration of copyright in the authorized bodies of Kazakhstan [50].

Based on the developed software systems, the operation of structures at all stages of the life cycle is studied. Various effects of the influence of nonlinearities on the behavior of structures are revealed. A study established the dependence of the equivalent parameters of elasticity on the level of loading, the percentage of reinforcement, the cracks formation scheme, and other factors [44].

Solutions for the development of practical recommendations have been obtained on the effective calculation of composite shells and plates in a nonlinear formulation in the concept of European standards.

**Figure 3:**
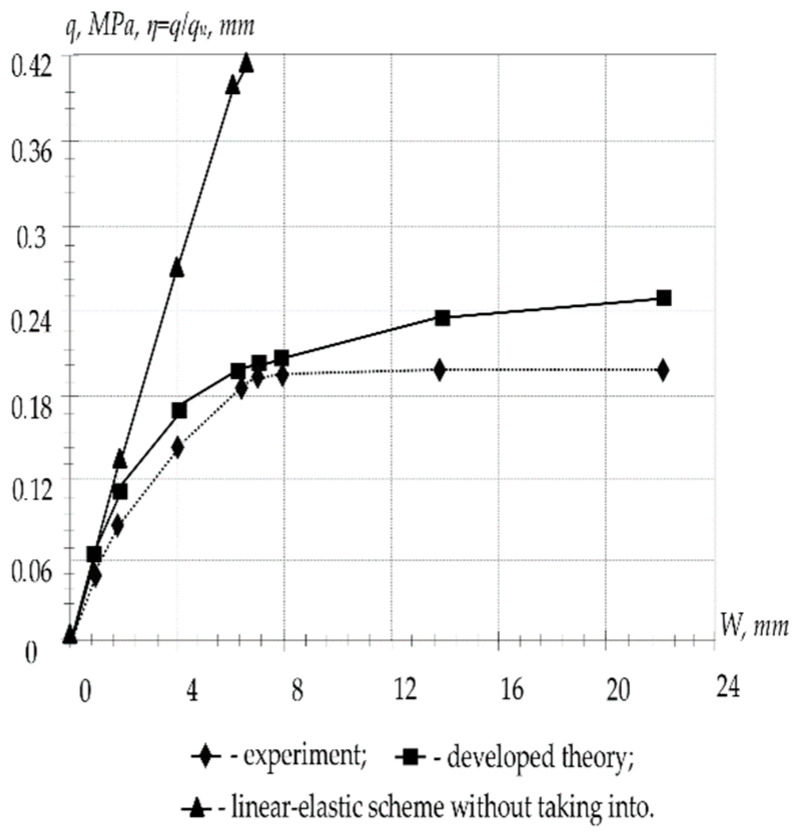
Load–deflection dependence graph for plate P-1.

Figure 3 shows a graph of the build-up deflection at the center of the plate. The results of the calculation of the plate according to the linear elastic scheme are presented without taking into account crack formation, according to the proposed theory and also according to experimental research. An insignificant discrepancy between the calculation results according to the proposed theory and experimental data (7–12%) confirms the reliability of the developed nonlinear calculation theory. Taking into account the physical nonlinearity gives a significant correction in bearing capacity values for plates with high content of reinforcement.

Short-term ultimate loads for P-1 with the following parameters: a=b=55 cm, s=a/b=1, 2h=4 cm, $h_{a1}=h_{a2}=h=1.8$, $F_{a1}^{\prime }=F_{a2}^{\prime }=0;\;F_{a1}=F_{a2}=0.0707$, $E_{0}=3.3\cdot 10^{4}$ MPa, $E_{a}=1.7\cdot 10^{5}$ Mpa, $v_{0}=0.2$, $R_{cub}=24.3$ Mpa, $R_{cs}=14.5$ Mpa, $R_{ts}=1.45$ Mpa, and $R_{a}=327.6$ MPa.

**Figure 4:**
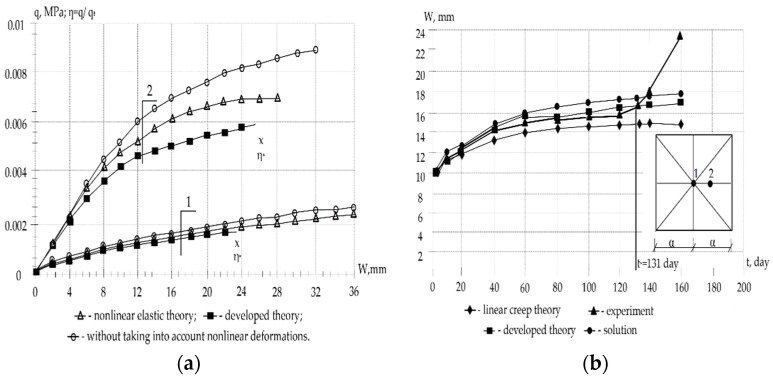
Dependency graph: (**a**) load–deflection, (**b**) deflection period: 1—shell S-1 and 2—shell S-2.

Figure 4 shows the results of the calculation of reinforced concrete shells according to a linear elastic scheme without taking into account cracks and also taking into account crack formation and the inelastic properties of materials.

In Figure 4a, the triangles show the curves of a numerical and experimental study of the behavior of shells O-1 and O-2 according to a nonlinear elastic scheme. A comparison of them with the rectangle curve obtained according to the proposed theory shows their insignificant discrepancy for particularly flat shells and more significant discrepancy for shells with a large rise. Circles represent curves calculated without taking into account the nonlinear components of deformations and crack formation. The higher the lift and the load level, the greater the discrepancy in the values (and other parameters) obtained from the linear and nonlinear theory of elasticity, taking into account plasticity and crack resistance.

The general figures and explanations for Figure 4b are summarized in a Table 1.

The following initial parameters are set: a=b=100 sm, s=a/b=1; 2hh=0.33 sm, $h_{01}=h_{02}=h=0.335$ sm, $F_{a1}^{\prime }=F_{a2}^{\prime }=0;\;F_{a1}=F_{a2}=0.00904$ sm, $E_{0}=2.4\cdot 10^{4}$ MPa, $E_{a}=2.1\cdot 10^{5}$ MPa, $v_{0}=0.2$, $R_{cub}=22.1$ MPa, $R_{cs}=13.2$ MPa, $R_{ts}=1.32$ MPa, $R_{a}=310.0$ MPa, $\sigma_{i}=\varepsilon_{i}$—cubic dependence, $A_{3}=1.1753902\cdot 10^{10}$ MPa, $k_{1}=k_{2}=0.55\cdot 10^{-3}$ sm at (0–1); $k_{1}=k_{2}=1.1\cdot 10^{-3}$ sm at (0–2); $\varphi \left(t\right)=\varphi_{\infty }\left(1-\beta_{1}e^{-\gamma_{1}\cdot t}-\beta_{2}e^{-\gamma_{2}\cdot t}\right).$ Short-term critical loads $q_{t}=0.00175\;MPa\;for\left(S-2\right);\;q_{t}=0.0058\;MPa\;for\left(S-2\right).$ Long-term critical loads $q_{l}=0.0043\;MPa\;for\;(S-2)$ with creep parameters: $f\left(x\right)=1+\beta \sigma_{i};\;\sigma_{i}\left(t\right)\leq \eta \cdot R_{cs};\;\beta =\vartheta \cdot \left(\frac{\sigma_{i}\left(t\right)}{R_{пc}}-\eta \right);\;\sigma_{i}\left(t\right)=E_{eq}\left(t\right)\cdot \varepsilon_{i}\left(t\right);\;\varphi \left(t\right)=\varphi_{\infty }(1-\beta_{1}e^{-\gamma_{1}\cdot t}-\beta_{2}e^{-\gamma_{2}\cdot t});\;\varphi \left(t\right)=\varphi_{\infty }(1-\beta_{1}e^{-\gamma_{1}\cdot t}-\beta_{2}e^{-\gamma_{2}\cdot t});\;\varphi_{\infty }=0.52;\;\beta_{1}=1;\;\beta_{2}=0;\;\gamma_{1}=0.04;\;\vartheta =0.015;\;\eta =0.35.$ As an example, models of flat reinforced concrete shells were calculated, the characteristics of materials and geometric dimensions of which are taken in accordance with the data of experimental studies performed by A.A. Oatul and A.P. Novoselov [49] (shell O-1 at $k_{1}=k_{2}=0.55\cdot 10^{-3}$ cm, O-2 when $k_{1}=k_{2}=1.1\cdot 10^{-3}$ cm and O-2D); I.A. Suslov [48] (plate P-1), as well as in NIIZHBA under the leadership of G.K. Khaydukov and V.V. Shugaev [47].

A comparison of the values of short-term limit loads for the shell and plate under consideration, calculated with and without taking into account the nonlinear properties of concrete, is shown in Table 1.

At the same time, short-term limit loads P-1 have the following parameters: a=b=55 sm, s=a/b=1; 2h=4 sm, $h_{01}=h_{02}=h=1.8$ sm, $F_{a1}^{\prime }=F_{a2}^{\prime }=0;\;F_{a1}=F_{a2}=0.0707$ sm, $E_{0}=3.3\cdot 10^{4}$ MPa, $E_{a}=1.7\cdot 10^{5}$ MPa, $v_{0}=0.2$, $R_{cub}=24.3$ MPa, $R_{cs}=14.5$ MPa, $R_{ts}=1.45$ MPa, and $R_{a}=327.6$ MPa.

Numerical studies have shown that according to the linear elastic scheme, the calculation of the instantaneous critical load $q_{M}\;$gives inflated values up to 56%; according to the linear theory of concrete creep, without taking into account cracking, the calculation of the long-term critical load $q_{д}$ overestimates its value up to 39%. With an increase in the lifting of the shell, the correction made taking into account the nonlinearity of concrete deformation is most significantly manifested. The dependence of the ratio of the long-term critical load to the corresponding short-term $\alpha_{0}=q_{д}/q_{M}\;$on: parameters of plasticity, creep, cracking, geometric characteristics, initial deflection, support conditions, etc., is revealed.

As a result of numerical experiments, the dependences of equivalent elasticity parameters on the loading level of ${\;E}_{эк}\left(\eta \right),\;{\;\upsilon }_{эк\;}(\eta )$, the percentage of reinforcement of $E_{эк}\left(\mu \right),\;\upsilon_{эк\;}\left(\mu \right)$, schemes of crack formation and development, the redistribution of forces, changes in stiffness, etc., are established.

## 4. Conclusions

A deformation model of a section with cracks and inelastic properties of materials has been created. A new nonlinear theory for calculating a reinforced concrete shell is proposed based on the replacement of the original shell with a continuous equivalent elastic shell. The behavior of the shell over the entire range of loading and existence is investigated by solving resolving systems of differential equations of equilibrium, motion, and perturbation of an equivalent structure. New scientific results on the work of inelastic composite shell structures have been obtained, and they are in good agreement with the known solutions [46,47] and experienced data [48,49]:

- To research the stability of an equilibrium state, it is not enough to use geometrically and physically nonlinear dependencies, based on the nonlinear theory of elasticity. Calculation of short-term and long-term critical load according to the linear elastic and nonlinear elastic scheme in comparison with the developed theory gives an overestimation of the values $q_{s}$ and $q_{l}$;
- The linear elastic calculation scheme overestimates the values $q_{s}$ up to 56%; calculation according to the linear theory of creep without taking into account crack formation overestimates the values $q_{l}$ up to 39%;
- The installed dependency $\alpha =q_{l}/q_{s}$ from the load level, the parameters of plasticity, creep, reinforcement percentage, the crack formation scheme, geometric characteristics, initial deflection support conditions of support and other factors;
- Taking into account that physical nonlinearity gives a significant correction in the values of the ultimate load $q_{u}$ for shells with high lift and reinforcement content, for very shallow shells, it is essential to take geometric nonlinearity into account.

Numerical studies are given on the basis of the proposed theory, algorithms, and software package. The results obtained in the course of numerical experimental studies have an insignificant error of 5–10%, which is in satisfactory agreement with the data of field experiments on the models of other authors. Consequently, the application of the theory gives quite acceptable results in solving problems of the theory of concrete creep.

The developed nonlinear theory of calculation of composite physically and geometrically nonlinear thin-walled systems can be applied to the calculation of structures made of concrete, metal, and other structural materials operating in a complex stress state at real loading levels, as well as in conditions of nonlinear creep of the material and the presence of cracks.

Other possible areas of coverage of the proposed theory can be the calculation of reinforced shells and plates on a nonlinearly deformable base, the calculation of reinforced concrete prismatic systems, the calculation of shells and plates of through-sections made of structural materials under conditions of nonlinear deformation and creep, and the calculation of reinforced shells and plates reinforced with stiffeners.

The circumstances listed above show that the codes of reinforced concrete structures must be brought into line with the principle general nonlinear theory of the calculation of structures (European standard), which will require significant material costs and organizational efforts [51]. However, this is justified, as it facilitates the flow of foreign investment into the country, improving the quality of manufactured materials and structures and objects under construction, which means improving the quality of life of people [52,53,54].

## Figures and Tables

**Table 1:** Results of numerical research on the nonlinear calculation of reinforced concrete shallow shells and plates.

Type of Calculation	P-1		S-1		S-2	
	Critical Load Value, MPa	Comparati-Ve Assessment, %	Critical Load Value, MPa	Comparati-Ve Assessment, %	Critical Load Value, MPa	Comparati-Ve Assessment, %
**Short-term**						
Elastic-linear scheme	0.046	254.9	0.0022	100	0.0087	100
Developed theory	0.02	114.3	0.00175	79.6	0.0058	66.7
Experiment [48]	0.0185	100				
**Long** **-** **term**						
Method of calculation [47]					0.0027	71.1
Developed theory for linear creep:						
excluding cracks			0.00162	100	0.0056	147.4
taking into account cracks formation			0.00151	93.2	0.0047	123.7
The developed theory takes into account nonlinear creep:						
according to the criterion [29]			0.00146	90.1	0.0043	113.2
Experiment [49]					0.0038	100

## Data Availability

The data presented in this study are available on request from the corresponding author.
